# Experts Teaching Wound Management—A Survey of Current Practices in Germany

**DOI:** 10.1111/iwj.70694

**Published:** 2025-05-26

**Authors:** Lilian Mahler, Pernilla Virginia Conrad, Dorothee Busch, Cornelia Erfurt‐Berge

**Affiliations:** ^1^ Department of Dermatology Uniklinikum Erlangen, Friedrich‐Alexander‐Universität Erlangen‐Nürnberg (FAU) Erlangen Germany; ^2^ Department of General, Visceral, Thoracic, Transplantation and Pediatric Surgery University Medical Center Schleswig‐Holstein, Campus Kiel Kiel Germany

**Keywords:** interprofessional, leg ulcer, medical student, wound care

## Abstract

This study aimed to gain insights into wound care education for medical students in Germany. An exploratory study with an online survey was conducted among wound experts of different professions, requesting details about educational programmes concerning teaching content, methodology, integration into current curricula and teaching faculty. The analysis included 118 datasets gathered from 17 doctors, 93 nurses and eight other health professionals. In 48.9% of cases, wound management was taught by different health care professionals, with doctors and nurses building a teaching team most frequently (68.2%). In about half of the cases, the medical students were learning together with trainees from other health professions. The teaching team was interdisciplinary in 40.4% of the courses. The large range of medical disciplines in which wound care was taught shows how variably this topic can be used in medical teaching. Nevertheless, the results from this survey also show that the topic is still clearly underrepresented in medical training, even though there is a high level of interest among medical students and a clear relevance for everyday practice as a physician. The medical teachers in this survey suggest more practical sessions on causal therapy, wound pain, quality of life and local wound care in the regular medical curriculum.

**Summary**

- Wound care is an underrepresented topic in medical curriculum.
- There is a number of different local teaching concepts but not anchored within the compulsory curriculum.
- Wound care can be excellently taught and learned by different healthcare professions in an interprofessional team.
- Medical students are highly interested in learning about wound care.

## 1 Introduction

The prevalence of patients with chronic wounds requires a solid interdisciplinary and interprofessional approach to wound management. Chronic wounds can affect patients of different ages and populations, and they include a variety of different causative diseases presenting with wounds. It is evident that the care provided to this patient group frequently does not meet the required standards. There is often a lack of basic care measures and diagnostic clarification, and referral to a specific centre occurs too late [1]. Potential reasons for this could be deficiencies in the training of medical students with regard to wound care. While there are existing curricula for nursing students [2] and graduated physicians [3] as well as recommendations for educational qualification frameworks published by international wound healing societies [2, 4, 5], recommendations for medical students remain a rarity [6]. However, there is a need for medical students to acquire clinical skills in this area. Wound management can be taught by various didactic methods, including bedside teaching, simulations and role‐playing, which can effectively enhance both cognitive and practical skills [7]. Despite this, the current situation regarding wound management education for medical students reveals significant gaps in training. The majority of medical students report limited experience, often consisting of short, isolated, often theoretical sessions about wound care. A comparative analysis of medical schools in the United States, the United Kingdom and Germany found that only a small number offered specific elective courses in wound care [8]. In addition, a national study in the United Kingdom revealed a strong demand for more comprehensive wound management education, with only around 30% of students receiving clinically relevant content [9]. This lack of structured, in‐depth training contributes to students feeling unprepared to effectively manage patients with chronic wounds [10] and highlights the need for enhanced curricula that incorporate practical skills and interdisciplinary approaches. The integration of wound management into medical curricula is crucial, as it prepares future practitioners in all medical disciplines to navigate complex cases and refer patients to appropriate specialists in time [11]. Based on the topic of wound care, the importance of interprofessional collaboration in patient care can be well transferred in teaching concepts for medical students. In order to better assess the need for further measures, the purpose of our study was to perform an exploratory study with the aim to collect detailed data among experienced wound experts about wound care education for medical students in Germany and to elaborate further need for improvement. The approach encompasses the type of teaching content, methodology, teaching faculty and students' feedback.

## 2 Materials and Methods

We created a questionnaire comprising questions about (a) demographics of the participants in the survey and their teaching involvement, (b) specific questions about the offered teaching content for wound management (interdisciplinarity, interprofessionality), (c) size and duration of the teaching event, details of the scenario (classical lecture, hands‐on exercise, etc.), study year of the teaching offer, (d) content of the courses. By asking for interprofessional aspects within the teaching settings, we focused on different health care professionals (physicians, nurses, physiotherapist) and by interdisciplinary, we aim at the involvement of different medical disciplines (e.g., surgery, dermatology etc.). This was clarified within the questionnaire.

The questions of the survey were elaborated by an interdisciplinary team of physicians (surgery, dermatology) with a background both in medical education and wound care. A medical student and a wound specialist nurse were asked to contribute to the questionnaire. At the end of the survey, the respondents were asked for their personal recommendation concerning (i) future teaching topics, (ii) preferred type of teaching (lecture, seminar, practical course), (iii) favoured study year to teach contents about wound management. Additionally, we asked the participants for their personal assessment of the extent of teaching programmes for medical students in the field of wound management in general and the students' desire to learn more about this topic. All questions were focused on wound care education for medical students. Teaching offers for nursing or other healthcare professionals only were not involved.

The questionnaire sheet was digitised with SoSci Survey Version 3.5.07 [12]. All participants gave their informed consent before starting the survey. An ethics vote was not required due to a completely anonymous setting of the survey and analysis, according to informal consultation with the local ethics committee. Descriptive data only are shown as results. Due to the exploratory design of the study, no statistical analyses were performed. Information about the project and access to the online survey was distributed via the associated journal of the ‘Initiative Chronische Wunden e.V. (ICW)’, Germany's largest Wound Management Society, and via ICW social media channels. By this way we were able to reach a group of experts who can be assumed to have a certain knowledge about wound care, even if it was not possible to specifically address those who are personally involved in wound care education for medical students. Participants were given the opportunity to voluntarily contact the project team via email after the study for further networking. The questionnaire has been piloted for understandability and comprehensiveness within a group of wound care specialists from different disciplines and professions in advance of the digital roll‐out. It may be important to note that in Germany, nurses´ or other health professionals´ education is not academicised which means not offered in an university setting. Therefore our work focuses on curricula for medical students (e.g., future physicians), although in some scenarios they are taught together with other professions.

## 3 Results

In the period from June to October 2024, a total of 254 participants took part in the survey. Some respondents cancelled the survey prematurely. We therefore determined a cut‐off question at the end of the epidemiological part of the questionnaire to define an analysable dataset. Finally, we were able to quantitatively and qualitatively analyse 118 datasets, including 49 fully completed datasets with all 32 answers possible. The individual number of total responses is therefore also stated for each result as *n*_tot_.

The group of participants is characterised as shown in Table 1. Among the 17 physicians, nine work in dermatology, six in surgery (e.g., trauma surgery, visceral surgery, vascular surgery) and two in internal medicine (e.g., diabetology, angiology). In addition to these disciplines already mentioned, the participating nurses work in general medicine, neurology and neuropaediatrics as well as psychiatry.

**TABLE 1:** Socio‐demographic data of participants.

**Profession**	
Physicians in total	17 (14.41%)
Residents	3 (17.6%)
Specialist physicians	4 (23.5%)
Senior consultants	8 (4.0%)
Chief physicians	2 (11.8%)
Physicians with pertinent formal qualification in wound management (e.g., “Ärztlicher Wundexperte ICW”)	9 (52.9%)
Physician with teaching assignment	11 (64.7%)
Medical specialties	
Surgery	6 (35.2%)
Dermatology	9 (52.9%)
Internal Medicine	2 (11.8%)
Nurses	93 (78.81%)
Other professions^a^	8 (6.78%)
Nurses or other professions with pertinent formal qualification in wound management (e.g., ‘pflegerischer Wundexperte ICW’)	92 (91.1%)
**Professional field**	
Clinic	72 (61.0%)
Maximum care clinic	32 (44.4%)
Specialised clinic	17 (23.6%)
Basic care clinic	23 (32%)
Medical practice	8 (6.8%)
Nursing facility	7 (5.9%)
Outpatient nursing care	15 (12.7%)
Others^b^	16 (13.6%)

^a^ For example, homecarer, physical therapist, medical student.

^b^ For example, medical supply shop, educational centre, physical therapy practice.

Forty‐four respondents are generally involved in the training of medical students as teachers. Among them are 15 physicians.

Fifty‐eight of our datasets are categorised as external reports, as the survey participant is not directly involved in the wound course, but the course exists at their institution. The other 60 interviews are self‐reports, as the survey participant contributes directly to the course as a teaching person.

### 3.1 Interprofessionality

In 51.1% (*n* = 46; *n*_tot_ = 90) of the reported teaching events, the teachers come from one medical profession. Of these, 37% (*n* = 17; *n*_tot_ = 46) are taught by physicians only, while 63.0% (*n* = 29; *n*_tot_ = 46) by nurses or other professions as a single teaching profession. The other 48.9% (*n* = 44; *n*_tot_ = 90) of the reported teaching events are performed by an interprofessional teaching team. In 68.2% (*n* = 30; *n*_tot_ = 44) of these interprofessional events, physicians and nurses act together as instructors, while in 13.6% (*n* = 6; *n*_tot_ = 44), the teaching team is made up of nurses and therapists from other professions like occupational therapists, physiotherapists, medical students, pharmacists or hygiene specialists. In 18.2% (*n* = 8; *n*_tot_ = 44) of all interprofessional courses, a multidisciplinary teaching team consisting of physicians, nurses and other professions is established.

Concerning the learners, in 49% (*n* = 49; *n*_tot_ = 100), the teaching offer is aimed at participants of different professions. Medical students together with nursing students are addressed in 76.6% (*n* = 36; *n*_tot_ = 47) of these interprofessional learning events. Among all learners, there are additionally 29.8% (*n* = 14; *n*_tot_ = 47) therapists in training or physicians at the beginning of their residency. In addition, 29.8% (*n* = 14; *n*_tot_ = 47) of the learners are therapists in training or doctors at the beginning of their residency.

### 3.2 Interdisciplinarity

In 40.4% (*n* = 36; *n*_tot_ = 89) of teaching offers, instructors from different medical specialties are part of the teaching team. These include, for example, specialist combinations from anaesthesia, nephrology, diabetology or forensic medicine, dermatology, surgery, oncology, pathology and internal medicine. Table 2 shows the variety of disciplines involved in teaching wound management. In 11.2% (*n* = 10; *n*_tot_ = 89) of teaching courses, the offer is coordinated under the leadership of more than one specialty.

**TABLE 2:** Potential specialties teaching wound management.

Specialties	Sub‐specialties
General medicine	
Anaesthesia	
Surgery	
	General surgery
	Vascular surgery
	Oral and maxillofacial surgery
	Plastic surgery
	Thoracic surgery
	Trauma surgery
	Visceral surgery
Dermatology	
Geriatrics	
Gynaecology	
Internal medicine	
	Angiology
	Diabetology
	Gastroenterology
	Cardiology
	Nephrology
	Rheumatology
Intensive care medicine	
Neurology	
Emergency medicine	
Onkology	
Orthopaedics	
Pathology	
Paediatrics	
Forensic medicine	
Urology	

### 3.3 Group Size and Teaching Formats

The offered courses comprise very different group sizes, including individual lessons in 3.8% (*n* = 3; *n*_tot_ = 78), small groups up to 10 students in 55.1% (*n* = 43; *n*_tot_ = 78), group lessons from 10 up to 50 students in 47.4% (*n* = 37; *n*_tot_ = 78) or over 50 learners in 3.8% (*n* = 3; *n*_tot_ = 78) of the courses described. Some of the courses combined different‐sized sessions.

In 28.9% (*n* = 22; *n*_tot_ = 76), the courses were part of the regular study curriculum while in 71.1% (*n* = 54; *n*_tot_ = 76), they were elective courses.

The standard medicine study programme in Germany is parted in four semesters of pre‐clinical studies and six semesters of clinical studies followed by a practical year. Some faculties offer longitudinal programmes with a more intensive focus of early patient contact. However, all students finish their pre‐clinical studies with a first exam (‘Physikum’). Although the teaching content is specified by a national catalogue, the exact time at which a subject is taught is not and is left to the individual university. Additionally, wound care is mainly subsumed under superordinate disease sections in this catalogue. In 23.9% (*n* = 17; *n*_tot_ = 71) of cases, the wound‐specific teaching offers took place during the pre‐clinical study years (Semesters 1–4), while in 43.7% (*n* = 31; *n*_tot_ = 71), they were settled within the clinical study years. Wound management education was primarily part of the curriculum of the practical year (60.6%). The range of hours per semester expended on the wound‐specific teaching events given in the survey was 1–60 h/semester, with a median value of 8 h/semester. In 24.5% (*n* = 13; *n*_tot_ = 53), the wound‐specific topic is offered only as part of a superordinated curriculum, for example, one lecture about chronic wounds within a general lecture series of one discipline. In 75.5% (*n* = 40; *n*_tot_ = 53) of cases, the teaching event is an autonomous offer with reference only to wound‐specific topics.

**FIGURE 1:**
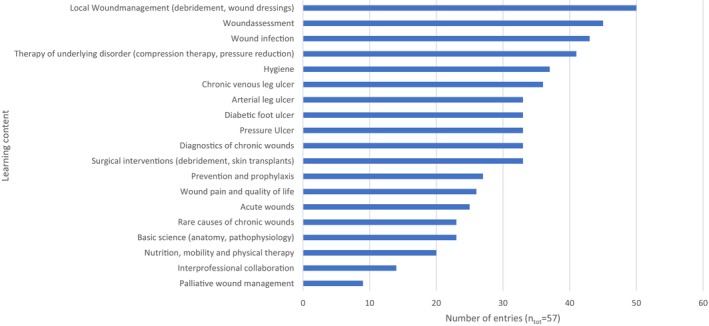
Teaching topics concerning wound management (multiple selection query from a suggestion list).

Table 3 shows the distribution of the different formats chosen for the teaching offer. In 44.8% (*n* = 26; *n*_tot_ = 58) of cases, a single format was chosen, while in 55.2% (*n* = 32; *n*_tot_ = 58), a combination of teaching methods was used. Figure 1 shows the topics chosen to be taught about wound care.

**TABLE 3:** Course formats for teaching wound management (multiple responses were possible).

Lecture	29.3% (*n* = 17; *n*_ges_ = 58)
Bedside teaching with patients	53.4% (*n* = 31; *n*_ges_ = 58)
Seminar	44.8% (*n* = 26; *n*_ges_ = 58)
Internship in a medical field with focus on patients with wounds	48.2% (*n* = 28; *n*_ges_ = 58)
E‐learning course	3.4% (*n* = 2; *n*_ges_ = 58)
Blended learning format	10.3% (*n* = 6; *n*_ges_ = 58)

### 3.4 Assessment and Recommendations for the Future

**FIGURE 2:**
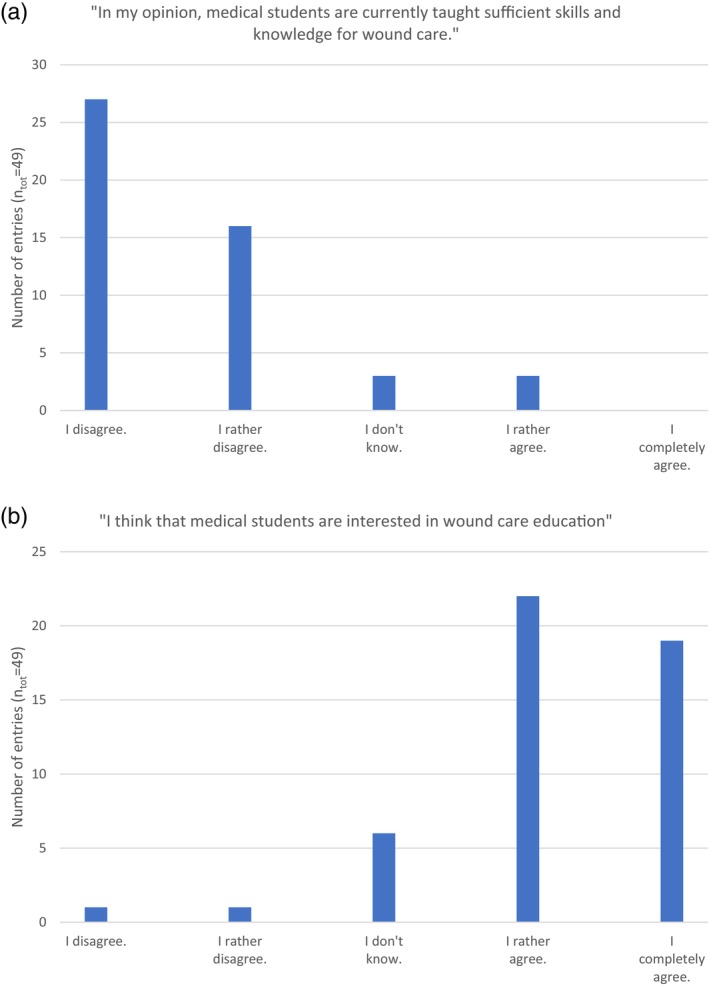
(a and b) Rating of statements concerning the extent of wound care education and the students′ attitude towards wound care education.

The respondents evaluated a given statement (‘The course was evaluated by the students as instructive and useful’) about their reported course in 52.8% (*n* = 28; *n*_tot_ = 53) with total agreement. In 24.5% (*n* = 13; *n*_tot_ = 53), they rather agreed, while in smaller rate they rather (3.8% (*n* = 2; *n*_tot_ = 53)) or totally (18.9% (*n* = 10; *n*_tot_ = 53)) disagreed. Figure 2a,b shows the respondents′ opinion about the current situation concerning wound care as an underrepresented teaching topic in medicine study programmes.

When asked for a learning goal in general concerning wound management courses, the answers comprised gaining a basic understanding of phase‐appropriate wound care and recognising underlying causes of wounds. Raising awareness of the needs of patients especially with chronic wounds was also mentioned as well as interprofessional and interdisciplinary approaches in wound care.

Apart from the topics actually taught in the courses described, the respondents consider the following five aspects to be particularly important for medical students: causal therapy (e.g., compression, pressure relief) (71.4%; *n* = 35; *n*_tot_ = 49); wound pain and quality of life (69.4%; *n* = 34; *n*_tot_ = 49); local wound therapy (69.4%; *n* = 34; *n*_tot_ = 49); diagnostics (69.4%; *n* = 34; *n*_tot_ = 49) and treatment of acute wounds (46.9%; *n* = 23; *n*_tot_ = 49). A total of 83.7% (*n* = 41; *n*_tot_ = 49) of the participants would find it useful to integrate the above‐mentioned teaching content more into the regular curriculum of the medical study programme and not just offer it as an optional elective course. When asked for their favourite time point to teach wound care within the medical study programme, the respondents preferred, with 63.3% (*n* = 31; *n*_tot_ = 49), the practical year (Semesters 11–12), or (with decreasing frequency) the Semesters 8–10. The recommended teaching formats were bedside teaching with patients (73.5% (*n* = 36; *n*_tot_ = 49)), interactive seminars (67.3% (*n* = 33; *n*_tot_ = 49)) or practice on models 51% (*n* = 25; *n*_tot_ = 49). Formats like classical lecture or exclusive e‐learning offers were less frequently considered suitable. The majority of respondents (79.6% (*n* = 39; *n*_tot_ = 49)) regarded it as important for students to be integrated into professional teams (e.g., interprofessional training wards, work shadowing, clinical traineeships) in future courses.

## 4 Discussion

The occurrence of acute or chronic wounds in particular can generally affect doctors from all clinical disciplines and requires certain basic knowledge. Enhanced education in primary care will improve uptake of evidence‐based practice [13]. However, this is currently not adequately taught in medical school, with this gap between needs and current education being present even at an international level [8, 9]. Our analysis shows that medical teachers are already aware of the problem of lack of training. However, they are still working with individual local solutions. Prevention and treatment of chronic wounds require an interprofessional approach [14]. Other healthcare professionals like nurses or physical therapists also play an important role in the care of people with chronic wounds and are often much better trained in this area. However, indications for diagnostic measures and therapeutic decisions remain the responsibility of the physician. An interprofessional collaborative practice can be implemented right from the start of graduate training [15]. Practice‐orientated learning environments help healthcare learners from different professional backgrounds to coordinate care plans in collaboration, for example, with patients and their families. Interprofessional education is regarded as essential to the delivery of coordinated health services that are safer, more effective, more efficient and more sustainable for patients.

While there are a number of interprofessional training concepts, for example, for trauma scenarios, only a few interprofessional educational concepts for wound care have been described so far for undergraduate medical students [16]. Bergendahl et al. [10] developed an interprofessional skills lab course where nursing students and medical students practiced on wound models in small interprofessional groups. In another concept described by Friman et al. [17], nursing and medical students participate in practical sessions and reflect together about their professional identity. Parker et al. [18] brought together nursing students, podiatry students, pharmacy students and exercise/nutrition science students in an 18‐h comprehensive programme with theoretical lectures, tutorials and clinical practice in wound scenarios. Even though the presented survey in our work specifically asked about programmes for medical students, the topic of wound care offers many options for integration into curricula, both interprofessionally (nurses, students, physicians, therapists) and at different stages of training (under‐ or postgraduate). Learners with different levels of knowledge and practical experience can contribute to learning success as peer teachers.

As shown by the number of respondents in our survey coming from a nursing background, the role of a nurse professionalist as a teacher for medical students is tremendous; although in Germany, the involvement of nurses as teachers for medical students is more informal and not a general concept. Also, therapists from other professions like physical therapy or nutrition science can be involved in such interprofessional teaching groups. When searching literature, it has to be taken into account that nurses in Germany are mainly not yet educated in an academic environment, but rather in the traditional way of a vocational training programme where wound care education is also implemented. Although in other European countries, content for wound care education for nursing students is clearly defined and curricula are implemented on a regular basis [19, 20], comparable studies on a similar scale for medical students are missing, not only in Germany [8]. There are only single educational interventions available in the literature [10, 21, 22], but no superordinate framework specifically concerning wound care. Jung et al. recently published a curriculum schema for chronic leg ulcers implemented at the University of Oregon [6]. As evidenced by our findings, the topic of wound care can be approached from a variety of perspectives within the medical field. Our results exemplify the integrative possibilities of a holistic, patient‐centred problem like a chronic wound into the curriculum of a diverse range of medical disciplines. In addition to the cognitive learning objectives pertaining to the pathophysiology and diagnosis of chronic wounds, practical skills such as the application of dressings, sonography and debridement procedures can be taught. This was also an important recommendation for teaching frameworks, according to the survey respondents.

Although several digital education programmes for wound care have been shown to be effective, the results from our survey emphasise the importance of practical experience on models, simulators or real patients [23]. Affective learning objectives can also be integrated into curricula in the domains of caring for people with chronic illnesses or the implementation of preventative measures. Due to the high level of practical relevance, the respondents in our survey saw such teaching concepts as being located preferably in late semesters or the practical year. However, some German universities already offer more longitudinal curricula for their students where wound healing processes can also be taught in pre‐clinical courses, for example, anatomy or physiology. The respondents concur that the subject of wound care is of significant interest to the students, yet acknowledge the necessity for further enhancement. The diagnosis of the underlying causes of chronic wounds and wound healing disorders, as well as their causal therapies, was identified as an important learning topic. This is in line with the identified shortcomings in the care of patients with chronic wounds [1]. The implementation of educational frameworks could therefore lead to improved patient care in the long term. However, for a long‐term anchoring of such programmes and a broad expansion of the topic, the programmes about wound care need to be embedded firmly in the compulsory medical curriculum and not just limited to elective courses as observed so far. Additionally, wound care education can be used to elaborate so‐called ‘spiral’ or longitudinal curricula where an early connection to wound care education can be made through different sciences and specialities, starting with the pathophysiology of wound healing in basic science to discussion about therapy in the clinical sciences later [24].

## 5 Limitations

### 5.1 Sample

The original sample of persons with access to the questionnaire were all wound society members. It therefore can be expected that the respondents are health carers with experience or increased interest in treating people with chronic wounds. Almost all participants had a special qualification in wound management. This can also be seen as a potential bias, as it can be assumed that only highly motivated people who are aware of the problem took part in the survey. Years of professional experience had not been queried in detail. There is also a number of what we called external reports from wound experts not involved directly in teaching medical students. Nevertheless, we have included this data in our analysis, as it can be assumed that respondents not directly involved in medical teaching still had insight into their institution's teaching structure.

### 5.2 Methods

Due to the technical requirements of the online survey, it was not possible to save data entry in‐between and to return to the questionnaire at a later date. This led to a decrease in the number of responses over the course of the questionnaire, as the questions were always presented to participants in the same order. As a result, a reduced number of analysable records for the last questions were available. Although the survey was initially distributed through ICW channels, it could potentially have been completed by non‐ICW members. Furthermore, it cannot be ruled out that a participant may have answered the survey more than once. Due to the nature of the online survey and since it has not been known in advance how many or which members of the society are also involved in wound care education for medical students, all members were given access to the survey. Projects outside of the ICW community may have been missed or underreported.

## 6 Conclusion

Wound care is a topic that can be effectively taught across a range of medical disciplines. It is suitable for basic science courses as well as for patient‐related training or simulations. At present, the topic is not sufficiently anchored in the mandatory medical curriculum, despite its high clinical relevance in later practice. It is therefore necessary to collect further data and information on existing teaching programmes about wound care and to scientifically investigate the effectiveness of the methods tested.

## Ethics Statement

The authors have nothing to report.

## Conflicts of Interest

Cornelia Erfurt‐Berge is board member of the ‘Initiative Chronische Wunden e.V’. The other authors declare no conflicts of interest.

## Data Availability

The data that support the findings of this study are available from the corresponding author upon reasonable request.
